# Highly Automated Dipole EStimation (HADES)

**DOI:** 10.1155/2011/982185

**Published:** 2011-03-06

**Authors:** C. Campi, A. Pascarella, A. Sorrentino, M. Piana

**Affiliations:** ^1^Dipartimento di Matematica, Università di Genova, 16126 Genova, Italy; ^2^Sezione di Fisiologia, Dipartimento di Neuroscienze, Università di Parma, 143121 Parma, Italy

## Abstract

Automatic estimation of current dipoles from biomagnetic data is still a problematic task. This is due not only to the ill-posedness of the inverse problem but also to two intrinsic difficulties introduced by the dipolar model: the unknown number of sources and the nonlinear relationship between the source locations and the data. Recently, we have developed a new Bayesian approach, particle filtering, based on dynamical tracking of the dipole constellation. Contrary to many dipole-based methods, particle filtering does not assume stationarity of the source configuration: the number of dipoles and their positions are estimated and updated dynamically during the course of the MEG sequence. We have now developed a Matlab-based graphical user interface, which allows nonexpert users to do automatic dipole estimation from MEG data with particle filtering. In the present paper, we describe the main features of the software and show the analysis of both a synthetic data set and an experimental dataset.

## 1. Introduction

Traditional dipole fitting of MEG evoked fields is a time-consuming procedure providing subjective results and requiring expert users for reliable source estimation; however, it is still largely used even for evaluating MEG inverse methods based on the distributed current assumption [1, 2] and, in any case, proved to be notably effective in the reconstruction of focal sources [3]. Estimating current dipoles from MEG data is in fact a hard task, as it involves solving several interacting problems such as model order selection (for determining the number of sources), nonlinear optimization (for estimating the source locations), and linear least-squares fitting (for calculating the dipole strengths). Most automatic algorithms for dipole estimation presented so far, and in fact even traditional dipole fitting, work under a couple of important approximations: (1) the number of dipoles is assumed to be fixed during the whole sequence, presence or absence of a given source being coded in the strength of the source itself; (2) the source locations are fixed in time. The second assumption is justified by physiological arguments, because a neural population hardly moves within the head. Also the first assumption appears to be reasonable; however, methods based on these assumptions can hardly discriminate nearby sources, even if they are not overlapping in time, because two dipoles placed at close distance will interact and produce spurious activity. Furthermore, in some cases, particularly when the number of sources is estimated from the data covariance matrix exploiting algebraic results [4], temporal correlation can prevent automatic algorithms from correctly recovering the neural sources.

In [5], we have described a source estimation method exploiting Bayesian filtering and random finite sets and based on a completely dynamical model, rejecting the assumptions (1) and (2) previously mentioned: the number of sources can change during the sequence, as well as the dipole locations. The number of active dipoles and their locations are estimated dynamically and updated at each time sample from the data. The method works by approximating with a particle filter, that is, a sequential Monte Carlo algorithm, the posterior densities involved in the Bayesian filter. In a couple of publications, we have discussed possible advantages and limitations of particle filtering for MEG, showing direct [6] and indirect [5] comparisons with other available methods.

In the present paper, we describe the use of the graphical user interface (GUI) we have developed for the particle filter, HADES (highly automatic dipole estimation). HADES is an open-source, freely downloadable, Matlab-based software. The purpose of the GUI is at least twofold: on one hand, we aim at sharing methods and results with other researchers in the field, who may have the chance to investigate by themselves the potential and limits of particle filtering; on the other hand, we aim at reaching a larger audience of neuroscientists who may be less curious about the methodological aspects but more interested in the possible applications. 

The paper is organized as follows. In Section 1, a nontechnical description of the methodological issues is presented. In Section 2, we provide details on the software, including supported data types, license details, and computational aspects. In Section 3, we follow step-by step the analysis of both a synthetic data set and an experimental dataset, so as to introduce the reader to the practical use of the interface. In Section 4, we briefly summarize the main features of the presented software.

## 2. Methods

The present section describes the computational algorithm at the basis of HADES and the way it has been implemented in the software. It contains three subsections: the first one describes the models adopted and the input data; the second one describes the particle filter and the run-time parameters necessary for the filter to run; the third one describes the estimation procedure and the output provided by HADES.

### 2.1. Model Assumptions and Input Data

HADES is based on a dynamical dipolar model of neural activations: at each time point, each active area is represented as a single current dipole. There is no prior assumption on the number of active sources, and there is no limit on the total number of neural sources; however, for computational reasons, we impose an upper bound to the number of simultaneous active dipoles.

HADES is based on a discretized source space: dipoles can take only a finite set of predefined possible locations. The main advantage of this approximation is that lead fields can thus be used to save computational time. Furthermore, the source space can be either the whole brain volume or else the cortical surface when available; to further increase localization accuracy, also an orientation constraint can be optionally used (although cortical constraints should be managed carefully, since there are neurophysiological situations where, using dipole fitting, they may lead to biased or wrong results).

All the source parameters are assumed to be dynamical parameters. The number of sources is a dynamical variable, to be estimated from the data. Sources are also allowed to move during time, that is, to jump between neighboring points of the source space.

Noise is assumed to have a Gaussian distribution. An estimate of the noise spatial covariance matrix can be either loaded or calculated; using such estimate corresponds to a prewhitening of the data. Alternatively, one can assume that noise is white Gaussian and calculate an estimate of the noise power.

**Figure 1:**
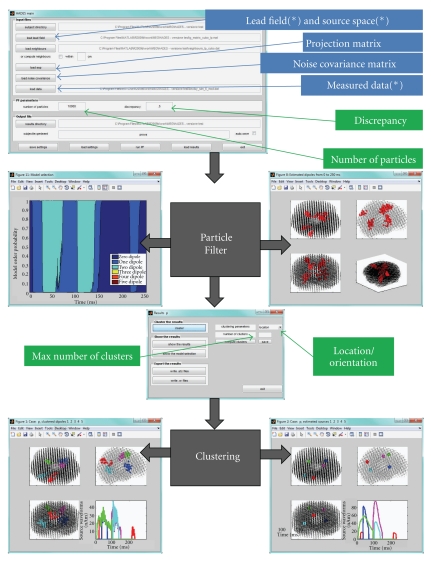
The input-output scheme of HADES: on top, the main window where the user can load input data (blue) and set the parameters (green); an asterisk indicates mandatory input data. The particle filter algorithm is presented as a black box giving two outputs (the model selection function and the estimated dipoles); the clustering algorithm assigns individual dipoles to clusters and computes the average location and the waveform of each cluster.

The input data needed to run HADES are therefore the source space and the corresponding lead field. The neighboring matrix, listing all the neighbours within a user-selected radius, is calculated by HADES. Optional inputs are the noise covariance matrix and a signal space projection matrix. See Figure 1 for a schematic representation.

### 2.2. Particle Filter and Run-Time Parameters

The core of HADES is a random finite sets (RFS) particle filter. Random finite sets are a mathematical tool for dealing with an unknown and varying number of objects [7]. Particle filtering [8] refers to an algorithm which tries a large number of dipole configurations, also called *particles*, choosing these configurations based on probabilistic criteria. The algorithm is sequential: it begins by analyzing the data measured at the first time point, *t* = 1, and proceeds time sample per time sample. At each time sample *t*, assume that a set of *N*_*p*_ dipole configurations is available; then the algorithm performs the following operations:

1. assign a weight to each dipole configuration, based on the difference between the measured data and the exact field produced by the dipole configuration,
2. use the cloud of weighted dipole configurations to calculate estimates of the number of sources and their parameters,
3. discard particles with low weights and multiply particles with high weights, in order to maintain only the most likely dipole configurations while preserving the total number of particles *N*_*p*_,
4. let each dipole configuration evolve randomly, thus producing the set of dipole configurations at time *t* + 1 needed at step 1, and start again from step 1.

According to the RFS framework, the number of dipoles in each particle may vary from zero to a maximum; dipole configurations may undergo loss or birth of dipoles during the temporal evolution at the fourth step.

 The number *N*_*p*_ of particles is the first parameter to set: using a large *N*_*p*_ guarantees in principle better results; the computational time is linearly increasing with this number, hence a good balance between stability and computational time has to be sought.

In the weighting procedure at step 1, the prior assumptions on the noise statistics play an important role, because the expected difference between the measured data and the exact field should be of the order of the noise. However, for several reasons the noise estimate can be unsatisfactory in many situations. In this case, one may want to have a weaker/stronger fit with the data, with respect to that provided by straightforward noise estimate. Therefore, we introduced the *discrepancy* parameter as a multiplicative factor for the noise estimate. Setting a small value (<1) for the discrepancy means that a stronger fit is required; the algorithm will then try to reproduce finer details in the data, possibly using a larger number of sources and possibly exhibiting a lower degree of stability and reliability. On the contrary, setting a large value means that a weaker fit is required, with the opposite consequence of ending up with a lower number of stable sources.

### 2.3. Source Estimates and Output

The estimation procedure in HADES goes through three main steps. The first two steps are performed at every sampled time point of an MEG sequence (step 2 in the previous subsection), and produce time-varying estimates of the dipole parameters: first, the algorithm obtains an estimate ${\hat{N}}_{t}$ of the number of dipoles and, then, calculates estimates of the actual parameters (location and dipole moment) for ${\hat{N}}_{t}$ dipoles. These dynamical estimates, however, do not identify individual neural sources in time, because there is no straightforward relationship between dipoles estimated and different time points. Given the collection of all dipoles estimated at all time points, a third step is then applied, which binds together dipoles estimated at different time points but possibly representing the same neural source. This clustering can be performed in two different configurations: either dipoles are grouped based only on their location, or else dipoles are grouped based both on location and orientation. The final number of clusters is estimated automatically with a recursive procedure, which starts from the user-defined maximum number of clusters and decreases this number until all the estimated clusters are significantly different. Once dipoles have been assigned to different clusters, likely corresponding to different neural sources, it makes sense to compute the average location of all dipoles belonging to each cluster; this average location can be considered as an estimate of the neural source location, and the corresponding source waveform can also be calculated.

The output of HADES consists in the dynamical estimates of the number of sources and of the source parameters, plus a global picture obtained from the clustering. Referring to Figure 4 as a typical result of a data analysis performed with HADES, the user can view the following.

1. The dynamical model order estimate (Figure 4(a)), that is, the posterior probability that the data have been produced by 1, 2, 3,… dipoles as a function of time; the cumulative distribution for the number of sources is visualized as an area, with different colors representing the probabilities of different models.
2. The dynamical estimates of the source locations (Figure 4(b)); while in this figure all the dipoles estimated in the whole sequence are superimposed, the user can in fact choose to visualize only the dipoles estimated in a selected time window.
3. The clustered dipole location estimates, with the corresponding amplitude waveforms (Figure 4(c)); since these waveforms are calculated for dynamical source locations, they exhibit a certain level of discontinuity in correspondence of jumps of the source location.
4. the average source location of each cluster (Figure 4(d)), with the corresponding amplitude waveform which is now continuous.

While the localization panels show the three standard views of the brain, figures contain in fact 3-dimensional information and the user can rotate the view.

## 3. Software Details

HADES is a Matlab-based graphical user interface, which needs Matlab to run. It has been written and tested under Matlab version 7.9.0, hence full compatibility is not guaranteed under earlier versions.

Input data can be provided in standard Matlab *.mat* format and in plain *ASCII* format; the Neuromag *.fif* format is supported through the set of functions contained in the MNE [9] Matlab toolbox http://www.nmr.mgh.harvard.edu/martinos/userInfo/data/sofMNE. More details on the format of input data can be found in the HADES manual, available at http://hades.dima.unige.it/. 

Results can be exported in different formats, for visualization in other toolboxes. At the moment, HADES features the following export options:

- a *.stc* file which contains the sequence of estimated dipoles in time, and can be visualized as a movie in MNE; furthermore, the very first time sample of the exported file contains the superposition of all the estimated dipoles, to get the overall picture of the estimated neural activity;
- a *.mat* file which contains the sequence of estimated dipoles in time and can be visualized as a movie in BrainStorm (http://neuroimage.usc.edu/brainstorm) again, the very first time sample of the exported file contains the superposition of all the estimated dipoles, to get the overall picture of the estimated neural activity;
- a *.w* file which contains the location of all dipoles estimated at all time points and can be visualized in FreeSurfer (http://surfer.nmr.mgh.harvard.edu/) [10, 11].

HADES is not bound to a specific hardware for MEG: all the hardware-dependent components are in fact contained in the input data (lead field, source space, and measurements). In principle, HADES may be applied to EEG data as well; experimental validation with electroencephalographic measurements is in progress.

The computational cost of the algorithm increases linearly with (i) the number of analyzed time samples and (ii) the number of particles. For running with 10,000 particles on a standard PC (CPU Intel Core2 Quad 2.83 GHz, RAM 4 GB) the algorithm takes on average 0.8 seconds per time sample.

HADES (http://hades.dima.unige.it/) is a free but copyrighted software, distributed under the terms of the GNU General Public Licence as published by the Free Software Foundation (either version 2 or at your option any later version).

## 4. Results

In this section we present two examples of source modeling performed using HADES. First, we use synthetic data so that the ground truth is known; the sample data analyzed here are contained in the HADES package for further analysis and testing. Then, we analyze an experimental data set corresponding to stimulation of left and right thumb.

### 4.1. Synthetic Data

**Figure 2:**
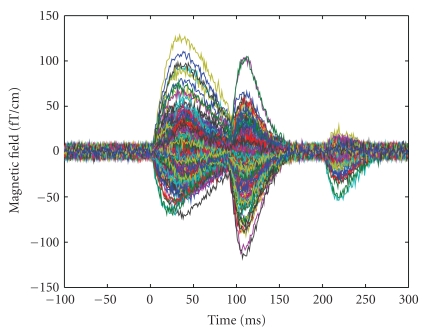
Synthetic data produced by the six sources described in Table 1 and Figure 3; only the signals from the gradiometers are shown.

**Figure 3:**
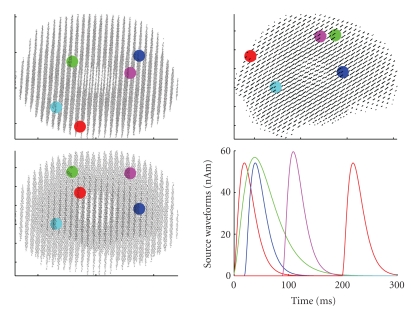
Location and dynamics of the 6 sources: the waveform of the cyan source is not visible as it is overridden by the time-correlated magenta source; the location of the yellow source is not visible as it is the same as the red source.

Data (see Figure 2) are produced by six sources: Table 1 summarizes locations and peak latencies of the sources, while Figure 3 shows both source locations and dynamics. Sources 2 and 3 have the same latency, but a different duration; Sources 4 and 5 have exactly the same waveform, that is, they are time correlated; Sources 1 and 6 are in the same location. The source points do not belong to the source space which is used by the inverse algorithm. MEG sensors correspond to the Neuromag Vectorview system which features 102 locations and 3 channels per location, one magnetometer and 2 planar gradiometers, for 306 channels. Here, we employ only the 204 planar gradiometers. White Gaussian noise is added: the noise standard deviation is 3 *fT*/*cm*; the SNR at the peak of the strongest source, calculated as 10 log _10_|*D*|^2^/|*N*|^2^, where *D* is the data matrix, *N* is the noise matrix, and |·| is the Frobenius norm, is about 10 dB. The superposition of all signals is shown in Figure 2.

We first load the source space and the lead field from the popup window. Then, we load the measurements: we set the starting time point (−100 ms), the sampling frequency (1,000 Hz), and the length of the prestimulus interval (from −100 to 0 ms) for estimation of the noise variance. The source space is formed by 13026 points with a regular spacing of 0.5 cm in the brain volume, and no cortical constraints are used.

#### 4.1.1. Single Run

We set the number of particles to 10,000 and the discrepancy parameter to 1 and run the particle filter.

**Figure 4:**
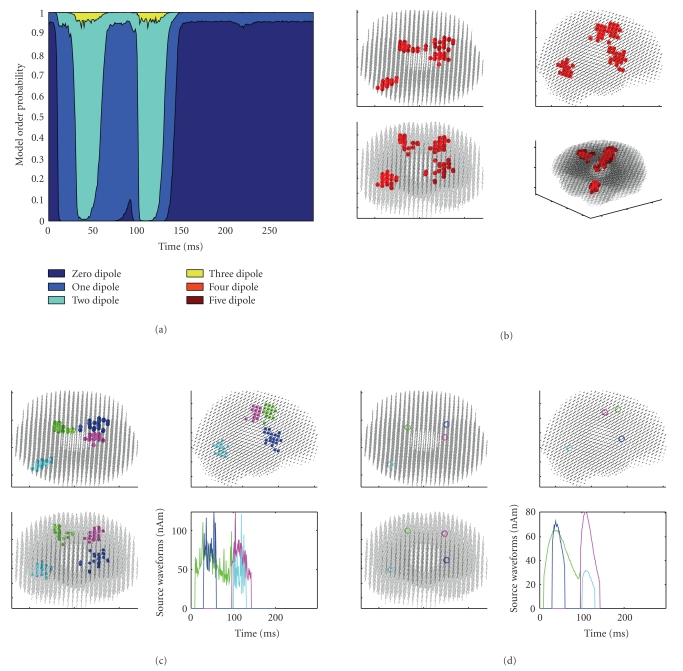
Results obtained with 10,000 particles and discrepancy 1. (a) Dynamical model selection function. (b) Superposition of all estimated dipoles at all time points. (c) Clustered dipoles and corresponding waveforms. (d) Average dipoles of the clusters in (c). Estimated dipoles show an expected spread around the true sources (cf Figure 3). From (b), it is evident that Sources 1 and 6, that is, the ones producing the weakest field, are not recovered. The model selection function indicates neural activity beginning at 10 ms (when the maximum probability switches from the zero-dipole model to the one-dipole model) and lasting until about 145 ms; in two time windows (30–55 ms and 105–130 ms), a two-dipole model is selected.

The results are shown in Figure 4. Two of the 6 sources producing the data are missing: in fact, they are the two Sources 1 and 6 in the same location, which are also the ones producing the smallest signal at the sensor level. The initial number of clusters was set to 4, due to both visual inspection of reconstructed dipoles (Figure 4(b)) and evidence from the model selection (Figure 4(a)), which indicates a two-dipole model in two separate temporal windows.

Considering all the reconstructed dipoles at all time points, the average distance between the dipoles and the corresponding sources is 1.1 cm, with a standard deviation of 0.8 cm, the maximum distance is 3.3 cm, and the minimum distance is 0.24 cm. Despite this large maximum error, the mean dipoles of the clusters (Figure 4(d)) appear to be good approximations of the true sources (cf Figure 3), featuring distances of 0.3 cm, 0.4 cm, 0.9 cm, and 1.35 cm from the true sources. This is explained as the estimated dipoles being quite symmetrically distributed around the true sources.

#### 4.1.2. Tuning the Parameters

**Figure 5:**
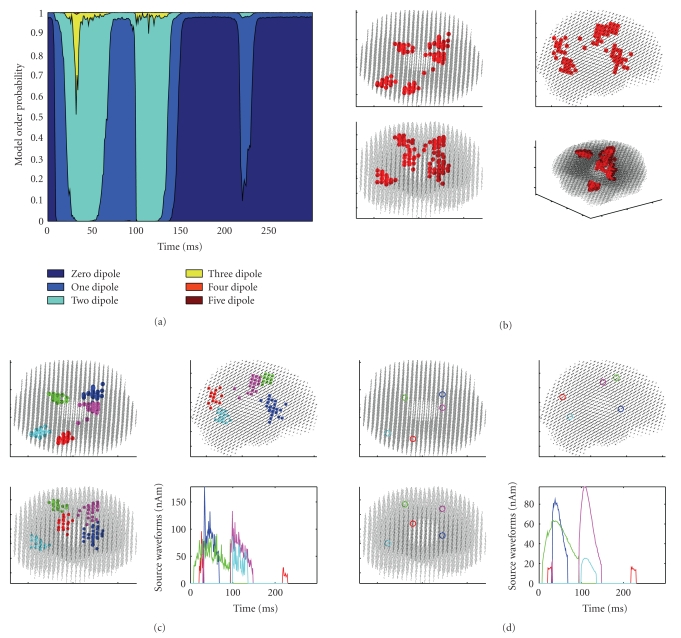
Results obtained with 10,000 particles and discrepancy 0.7. The model selection indicates now a one-dipole model in a short time window around 220 ms, corresponding to Source 6. In fact, both Sources 1 and 6 are now recovered correctly; the clustering procedure binds them in a single source, because they are exactly in the same location. The model selection also indicates that the two-dipole model is now selected for larger time windows with respect to the previous case with unit discrepancy; moreover, around 30 ms, the three-dipole model appears to have a nonnegligible probability, even though it does not exceed the 50%.

**Figure 6:**
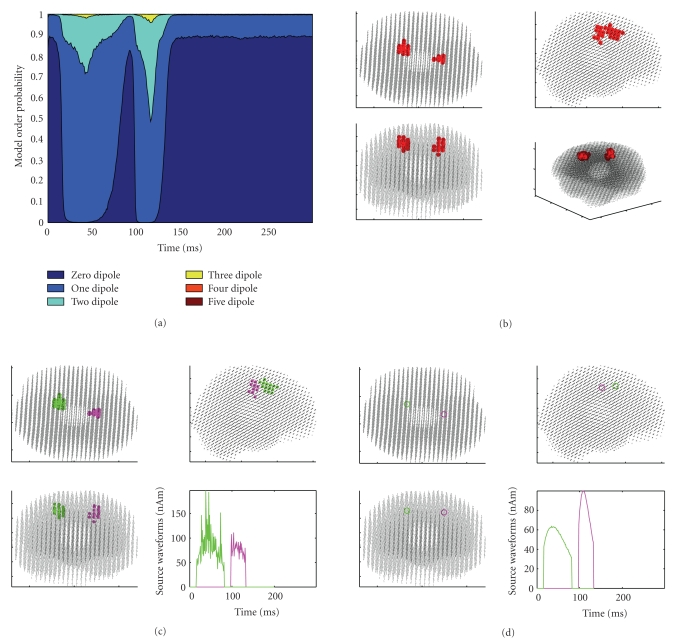
Results obtained with 10,000 particles and discrepancy 2. With this large value for the discrepancy parameter, the model selection in (b) exhibits lower probability for larger models, and the two-dipole model has nonnegligible posterior probability but is never the mode of the distribution. The set of estimated dipoles is now smaller and contains only two activations, corresponding to Source 3 and 4, that is, the two dipoles producing the strongest field.

As described in the previous section, tuning the discrepancy parameter corresponds to requiring higher/lower fit with the data. We run again the particle filter with 10,000 particles, first setting the discrepancy to 0.7 (higher fit required) and then to 2 (lower fit). The results are shown in Figures 5 and 6, respectively. With the lower discrepancy, the algorithm recovers also the two weaker sources, Source 1 and 6. The figure has been obtained by clustering the dipoles in 5 groups. With the higher discrepancy, the algorithm looses track of Sources 1, 2, 5, and 6.

Average distances between reconstructed dipoles and true sources are in the same range as for the unitary discrepancy.

### 4.2. Experimental Data

**Figure 7:**
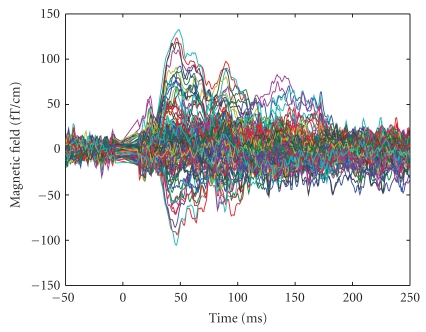
Averaged magnetic field for the stimulation of the left thumb.

**Figure 8:**
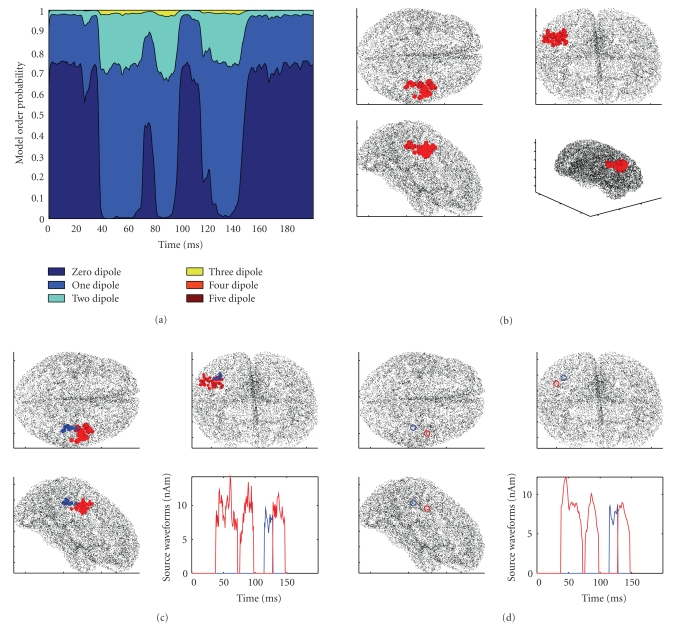
Left thumb stimulation; results obtained with 10,000 particles and discrepancy 1. The model selection in (a) indicates activity in a first time window beginning around 40 milliseconds after the stimulus and lasting until 100 ms and in a second time window between 115 and 140 ms. All estimated dipoles are in the right hemisphere, located around the somatosensory cortex. Clustering does not seem to add significant information to the estimated sources: the blue cluster is smaller and lasts few milliseconds.

**Figure 9:**
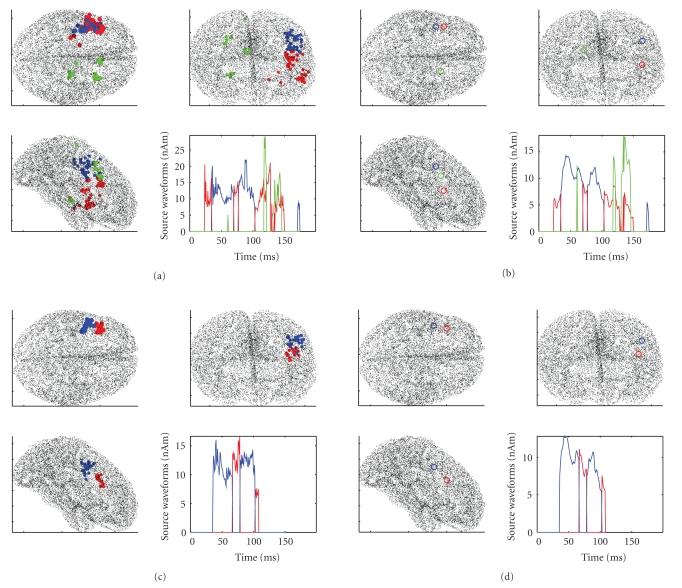
Right thumb stimulation. First row: results obtained with 10,000 particles and discrepancy 1. (a) shows the clustered reconstructions: most dipoles are located in the left hemisphere in proximity of the somatosensory cortex; however some reconstructions fall in the right hemisphere and are rather unstable. Arguing from such instability that the noise estimate was slightly too tight, we increased the discrepancy parameter to 1.5 to get the cleaner results of the second row.

MEG data were provided courtesy of Dr. Sabine Meunier (La Salpetriere Hospital, Paris), as made available for download on BrainStorm's website. The data were recorded on a CTF machine (151 axial gradiometers) at La Salpetriere Hospital, Paris. The protocol comprised shuffled electrical stimulation of the fingers from both hands; the analyzed data are averaged responses (400 trials) for the stimulation of the right thumb (R) and of the left thumb (L) (see Figure 7). The lead field matrix was exported using the BrainStorm software, as well as the source space; the source space consists of 15,010 source points distributed along the cortical surface. A distance of 1 centimeter was selected for calculation of the neighboring matrix. Both data sets were analyzed using 10,000 particles and the discrepancy parameter set to 1; the orientation constraint was not used, although available. Results for the left and right thumb stimulation experiment are described in Figures 8 and 9 respectively. With the left data, reasonable source localization is obtained with the first run, with the standard discrepancy value. With the right data, on the contrary, the standard parameter value provided reasonable localization in correspondence with the peak of activation, plus some other dipoles at later time points scattered in apparently less likely locations. Cleaner reconstructions can be obtained increasing the discrepancy parameter (see Figures 9(c) and 9(d)).

## 5. Discussion and Conclusions

HADES is a Matlab-based, freely downloadable software for dynamical estimation of current dipoles from MEG data. It is distributed under the GPL and has a simple graphical user interface, which allows nonexpert users to do dipole modeling automatically.

The particle filter HADES is based on [5] and tracks in time the posterior density for the dipole constellation; statistical estimators are used to provide dynamical estimates of the number of sources and of the source parameters. The main innovative feature of HADES, with respect to the available dipole estimation methods, is related to the underlying dynamical model: dipoles are not constrained to have a fixed position nor to be active for the whole time sequence. Instead, the number of sources and all source parameters are estimated at each sampled time point; in particular, HADES provides a dynamical model selection function, which indicates at each time point the probability that the data have been produced by 1,2,…, *N* dipoles. To obtain stable source estimates and continuous source waveforms, clustering procedures are implemented which bind dipoles representing the same source at different time points. Due to the generality of the underlying model, HADES can recover correlated sources and discriminate nearby dipoles with different orientations. On the other hand, the particle filter is more computationally demanding with respect to other estimation methods, and semianalytic solutions [12] to Bayesian filtering feature better statistical properties but higher computational requirements.

The performances of HADES were illustrated with a set of synthetic data produced by a complicated source configuration, as well as with a set of experimental data. Synthetic data were particularly useful to illustrate how the discrepancy parameter plays an important role in selecting larger/smaller number of sources. The same conclusion can be drawn also from the experimental data set, with the further consideration that in real situations the peculiar structure of neural noise is more likely to produce spurious activity.

The visualization of the results is limited to a very simple 3d plot of the source space with the estimated sources superimposed. However, the results can be exported for visualization in other toolboxes where superimposition onto high resolution MRI slices or inflated surfaces are possible. Export options to MNE, Freesurfer, and BrainStorm are supported at the moment. Forthcoming releases of the toolbox may feature better built-in visualization tools.

HADES has been thought as a highly specialized toolbox for dipole estimation. As such, it does not mean to replace other toolboxes but possibly to integrate with them to provide a different perspective on a data set. For this reason, no tools for multisubject analysis are under development at the moment, although HADES-reconstructed dipoles are saved in the.mat file of the results and can be utilized for statistical analysis by means of external toolboxes.

More in general, the toolbox is at its very first stage, and the development of the method will likely add more features to the toolbox. Possible future methodological developments include

- investigating strategies to remove spurious activations produced by neural noise,
- providing an estimate of the localization accuracy for each source, based on the spread of the underlying posterior density,
- modeling the neural sources as nondipolar currents, such as multipolar sources or cortical patches.

All future developments will head towards automation and reliability of source estimation from MEG/EEG data.

## Figures and Tables

**Table 1:** Parameters of the six sources used to simulate the data: source location, peak latency, and measured signal at the peak. Colors refer to Figure 3.

Source *n*	*x* (cm)	*y* (cm)	*z* (cm)	*t* (ms)	*fT*/*cm*
1 (red)	−1.37	−5.43	7.34	20	51
2 (blue)	3.74	4.54	5.66	40	57
3 (green)	−2.04	3.73	9.56	40	130
4 (magenta)	2.96	2.11	9.42	110	100
5 (cyan)	−3.43	−2.71	4.07	110	110
6 (yellow)	−1.37	−5.43	7.34	220	51
